# Clinical characterization of patients with primary aldosteronism plus subclinical Cushing’s syndrome

**DOI:** 10.1186/s12902-020-0490-0

**Published:** 2020-01-13

**Authors:** Shigemitsu Yasuda, Yusuke Hikima, Yusuke Kabeya, Shinichiro Iida, Yoichi Oikawa, Masashi Isshiki, Ikuo Inoue, Akira Shimada, Mitsuhiko Noda

**Affiliations:** 1 Department of Endocrinology and Diabetes, Saitama Medical University, Morohongo 38, Moroyama, Iruma-gun, Saitama, 350-0495 Japan; 2 Department of Home Care Medicine, Sowa Hospital, Sagamihara, Kanagawa Japan; 3 Department of Diabetes, Metabolism and Endocrinology, Ichikawa Hospital, International University of Health and Welfare, Chiba, Japan

**Keywords:** Primary aldosteronism, Subclinical Cushing’s syndrome, Adrenal tumor, Maximum tumor diameter, Diabetes mellitus, Serum potassium

## Abstract

**Background:**

Primary aldosteronism (PA) plus subclinical Cushing’s syndrome (SCS), PASCS, has occasionally been reported. We aimed to clinically characterize patients with PASCS who are poorly profiled.

**Methods:**

A population-based, retrospective, single-center, observational study was conducted in 71 patients (age, 58.2 ± 11.2 years; 24 males and 47 females) who developed PA (*n* = 45), SCS (*n* = 12), or PASCS (*n* = 14). The main outcome measures were the proportion of patients with diabetes mellitus (DM), serum potassium concentration, and maximum tumor diameter (MTD) on the computed tomography (CT) scans.

**Results:**

The proportion of DM patients was significantly greater in the PASCS group than in the PA group (50.0% vs. 13.9%, *p* <  0.05), without a significant difference between the PASCS and SCS groups. Serum potassium concentration was significantly lower in the PASCS group than in the SCS group (3.2 ± 0.8 mEq/L vs. 4.0 ± 0.5 mEq/L; *p* <  0.01), without a significant difference between the PASCS and PA groups. Among the 3 study groups of patients who had a unilateral adrenal tumor, MTD was significantly greater in the PASCS group than in the PA group (2.7 ± 0.1 cm vs. 1.4 ± 0.1 cm; *p* <  0.001), without a significant difference between the PASCS and SCS groups.

**Conclusions:**

Any reference criteria were not obtained that surely distinguish patients with PASCS from those with PA or SCS. However, clinicians should suspect the presence of concurrent SCS in patients with PA when detecting a relatively large adrenal tumor on the CT scans.

## Background

Primary aldosteronism (PA), an adrenocortical disorder caused by an adrenal tumor that overproduces aldosterone, accounts for 5 to 15% of patients with hypertension [1]. Cushing’s syndrome (CS), an endocrinopathy resulting from the prolonged, excessive adrenocortical secretion of cortisol, falls roughly into the following 2 categories: adrenocorticotropic hormone (ACTH)-dependent CS and ACTH-independent CS; the former includes Cushing’s disease that is primarily caused by a pituitary ACTH-secreting tumor and ectopic ACTH syndrome resulting from extrapituitary ACTH-secreting tumors (eg, bronchial carcinoid) [2], while the latter is usually caused by unilateral adenomas or carcinomas that provoke the autonomous adrenal cortical secretion [3]. Subclinical Cushing’s syndrome (SCS), an ill-defined endocrine disorder leading to the ACTH-independent secretion of cortisol from an adrenal adenoma that is not fully restrained by pituitary feedback [4], is known to cause hypertension, glucose intolerance, and dyslipidemia [5].

The concurrence of clinically overt hyperaldosteronism and subclinical hypercortisolism is rare in PA patients [6]. To date, a few number of studies have examined the clinicopathological features of patients with PA plus SCS (PASCS), the incidences of which have ranged between about 10 and 20% [7, 8]. Lower plasma ACTH levels and a greater tumor size were found in patients with PASCS than in patients with PA alone [8]. In the clinical settings, we rarely encounter PASCS patients who show a small adrenal tumor on the computed tomography (CT) scans and/or do not have a low plasma ACTH level in blood samples collected in the early morning. To examine the clinical features of PASCS patients in the present study, we compared clinical, laboratory, and imaging characteristics among patients with PA, SCS, or PASCS.

## Methods

### Patients

We conducted a population-based, retrospective, single-center, observational study in 187 patients (119 with PA, 54 with SCS, and 14 with PASCS) at Saitama Medical University Hospital, Saitama, Japan, between January 1999 and December 2016. Hypertensive patients with suspected PA or SCS, as well as normotensive or hypertensive patients with an adrenal incidentaloma were referred to our hospital. A total of 116 patients were excluded from the study: 31 who were diagnosed with PA or SCS only because tests required to definitely diagnose these endocrinopathies were not conducted; 61 who failed to meet the new Japanese diagnostic criteria of SCS [9]; 1 who failed to meet the new Korean diagnostic criteria of subclinical hypercortisolism [10]; and 23 who failed to meet the Japanese [11] and United States [12] diagnostic criteria of PA. Therefore, we investigated 71 patients who were definitely diagnosed with PA and/or SCS (45 with PA, 12 with SCS, and 14 with PASCS). This study was approved by the institutional review board of Saitama Medical University. Patients provided written informed consent to the use of their clinical and laboratory data in the study.

### Diagnosis of PA and SCS

Hormones required for the diagnosis of PA and SCS were assayed according to the procedures described in the pertinent guidelines [9, 11]. Serum cortisol and plasma ACTH levels were determined by electrochemiluminescence immunoassay, plasma aldosterone concentration (PAC) and plasma renin activity (PRA) by radioimmunoassay, and serum dehydroepiandrosterone sulfate (DHEAS) level by chemiluminescent enzyme immunoassay (SRL Inc., Tokyo, Japan). Blood samples were collected in the early morning (7 a.m. to 9 a.m.). PA was suspected when detecting elevated PAC (≥ 150 pg/mL), low PRA (≤ 1.0 ng/mL/hr), and/or the elevated aldosterone-to-renin ratio (> 200). We conducted the following 3 challenge tests in accordance with the Japanese guidelines of PA [11]: captopril challenge test, furosemide upright posture challenge test, and ACTH challenge test. PA was diagnosed when at least 1 of these 3 challenge tests afforded results compatible with the disease. Furthermore, we also referred to the American guideline of PA [12] for selecting only patients who met the diagnostic criteria for PA. Prior to the confirmatory tests, patients had not received any antihypertensive drugs for at least 2 weeks except for those with severe hypertension treated with calcium-channel blockers and/or α-blockers. Adrenal venous sampling (AVS), whose usefulness was well documented in the Japanese and United States guidelines [11–13], was conducted in all of patients who had PA or PASCS to make the differential diagnosis of uni- or bilateral aldosterone hypersecretion.

The low-dose (1-mg) dexamethasone suppression test (DST) and the corticotropin-releasing hormone (CRH) challenge test were conducted, and the diurnal rhythms of cortisol were also determined—all for the diagnosis of SCS. Moreover, the high-dose (8-mg) DST was also conducted to rule out ACTH-dependent CS. Test results were assessed in accordance with the diagnostic criteria advocated by the Japan Endocrine Society [9] to make the definite diagnosis of SCS. Concretely, patients were required to meet the requisites 1–3)—1) presence of an adrenal incidentaloma; 2) lack of characteristic features of Cushing’s syndrome; and 3) normal basal serum cortisol levels, as well as to have either of the requisites 4–6)—4) the cutoff value of serum cortisol level for the diagnosis of SCS was ≥ 5 μg/dL after the 1-mg DST, 5) the cutoff value of serum cortisol level for the diagnosis of SCS was ≥ 3 μg/dL after the 1-mg DST, and at least 1 of “Low plasma levels of ACTH in the early morning,” “No diurnal changes in serum cortisol levels,” “Unilateral uptake on adrenal scintigraphy,” “Low serum levels of DHEAS,” or the presence of “Transient adrenal insufficiency or atrophy of the attached normal adrenal cortex after removal of the adrenal tumor,” or 6) the cutoff value of serum cortisol level for the diagnosis of SCS was ≥ 1.8 μg/dL after the 1-mg DST, with the presence of “Low plasma levels of ACTH in the early morning” and “No diurnal changes in serum cortisol levels,” or the presence of “Transient adrenal insufficiency or atrophy of the attached normal adrenal cortex after removal of the adrenal tumor.” In the present study, we examined only patients who met the requisites 1–3) and either 1 of the requisites 4–6) as patients with SCS. All patients underwent 128-slice CT of the adrenal glands. ^131^I-adosterol adrenal scintigraphy was conducted in all of patients who had SCS or PASCS to specify the laterality of the adrenal tumor. Consequently, 7 of 12 patients with SCS and 8 of 14 patients with PASCS underwent adrenalectomy. Postsurgical histopathological examination confirmed cortisol hypersecretion based on the atrophy of the normal area adjacent to the adenoma of the removed adrenal gland [9].

### Study outcome measures

At the initial visit, all patients were checked up for their age and sex. Systolic blood pressure (SBP), diastolic blood pressure (DBP), and the outcome measures listed in Table 1 were examined in untreated patients. At the time of admission to the hospital for making the definite diagnosis, height and body weight were measured to calculate body mass index (BMI). In the early morning of the next day of admission to the hospital, blood pressures were measured. Blood samples were collected to determine PAC, PRA, as well as plasma ACTH, serum cortisol, and serum DHEAS levels. The laterality of the adrenal tumor was confirmed based on the results from AVS and/or CT. The Hounsfield number and MTD of adrenal tumors were determined on the CT scans.

**Table 1:** Clinical, laboratory, and imaging characteristics of untreated patients with PA, SCS, or PASCS

	Study diseases			*P* values in between-group comparisons			
	PA (n = 45)	SCS (n = 12)	PASCS^a^ (*n* = 14)	All^b^	PA vs. PASCS^c^	SCS vs. PASCS^c^	PA vs. SCS^c^
Clinical characteristics							
Age, y	56.2 ± 1.0	62.5 ± 12.6	60.8 ± 13.1	NS			
Sex, female, n (%)	29 (64.4)	7 (58.3)	11 (78.6)	NS			
BMI, kg/m^2^	24.9 ± 4.2	25.2 ± 5.9	26.0 ± 4.4	NS			
SBP, mmHg	165.6 ± 26.1	145.6 ± 26.9	150.1 ± 27.2	< 0.05	NS	NS	NS
DBP, mmHg	96.0 ± 13.6	80.0 ± 12.7	90.5 ± 16.4	< 0.01	NS	NS	< 0.01
Comorbidities							
Hypertension, n (%)	45 (100.0)	9 (75.0)	13 (92.9)	< 0.01	NS	NS	< 0.05
Diabetes mellitus, n (%)	6 (14.0)	6 (50.0)	7 (50.0)	< 0.01	< 0.05	NS	NS
Dyslipidemia, n (%)	25 (56.8)	10 (83.3)	9 (64.3)	NS			
Laboratory characteristics							
FPG, mg/dL	103.8 ± 28.5	150.0 ± 60.7	131.6 ± 52.1	< 0.005	NS	NS	< 0.01
HbA1c NGSP, %	5.7 ± 0.9	7.3 ± 2.2	6.5 ± 2.1	< 0.01	NS	NS	< 0.01
TC, mg/dL	197.5 ± 42.5	208.1 ± 54.7	195.1 ± 30.4	NS			
TG, mg/dL	131.7 ± 88.2	148.3 ± 52.9	141.3 ± 70.3	NS			
HDL-C, mg/dL	55.2 ± 15.3	53.6 ± 13.9	57.0 ± 12.4	NS			
LDL-C, mg/dL	117.6 ± 40.3	127.2 ± 47.5	112.9 ± 23.6	NS			
Serum potassium, mEq/L	3.3 ± 0.7	4.0 ± 0.5	3.2 ± 0.8	< 0.01	NS	< 0.01	< 0.01
Serum calcium, mg/dL	9.4 ± 0.4	9.5 ± 0.4	9.6 ± 0.4	NS			
Serum phosphorus, mg/dL	3.5 ± 0.5	3.3 ± 0.9	3.3 ± 0.4	NS			
UA, mg/dL	5.1 ± 1.1	4.9 ± 1.1	5.3 ± 1.3	NS			
Serum ALP, U/L	212.3 ± 46.3	256.8 ± 70.0	279.1 ± 105.4	< 0.005	< 0.01	NS	NS
Erythrocyte, 10^6^/μL	4.4 ± 0.5	4.5 ± 0.7	4.5 ± 0.3	NS			
Hemoglobin, g/dL	13.2 ± 1.6	13.7 ± 2.0	13.2 ± 1.4	NS			
Hematocrit, %	39.0 ± 4.1	41.3 ± 5.5	39.2 ± 4.1	NS			
Leukocyte, 10^3^/μL	5.8 ± 1.6	6.6 ± 1.3	5.7 ± 2.0	NS			
Neutrophil, %	61.7 ± 7.2	64.7 ± 12.7	63.7 ± 5.3	NS			
Lymphocyte, %	28.9 ± 6.8	27.2 ± 11.4	28.0 ± 6.2	NS			
Eosinocyte, %	3.9 ± 3.4	2.4 ± 2.1	2.4 ± 1.4	NS			
Imaging characteristics							
Laterality of tumor^d^							
Left-sided, n (%)	28 (62.2)	9 (75.0)	7 (50.0)				
Right-sided, n (%)	14 (31.1)	3 (25.0)	5 (35.7)				
Bilateral, n (%)	3 (6.7)	0 (0.0)	2 (14.3)				

Values are expressed as mean ± SD or count (%)

*PA* primary aldosteronism, *SCS* subclinical Cushing’s syndrome, *PASCS* primary aldosteronism plus subclinical Cushing’s syndrome, *BMI* body mass index, *SBP* systolic blood pressure, *DBP* diastolic blood pressure, *FPG* fasting plasma glucose, *HbA*_*1c*_ hemoglobin A_1c_, *NGSP* national glycohemoglobin standardization program, *TC* total cholesterol, *TG* triglyceride, *HDL-C* high-density lipoprotein cholesterol, *LDL-C* low-density lipoprotein cholesterol, *UA* uric acid, *ALP* alkaline phosphatase, *NS* not significant, *SD* standard deviation

^a^Patients with primary aldosteronism plus subclinical Cushing’s syndrome

^b^Analyzed according to the one-way analysis of variance

^c^Bonferroni’s correction was applied to the *p* values from Student’s t-test or Fisher’s exact test in multiple comparisons between two groups

^d^Determined by computed tomography, adrenal venous sampling, and ^131^I-adosterol adrenal scintigraphy

A value of *p* <  0.05 was considered statistically significant

The following terms were defined for PASCS: hypertension, SBP ≥ 140 mmHg and/or DBP ≥ 90 mmHg [14]; diabetes mellitus (DM), an fasting plasma glucose (FPG) level ≥ 126 mg/dL, a 2-h plasma glucose level ≥ 200 mg/dL in the 75-g oral glucose tolerance test, and/or a serum hemoglobin A1c (HbA1c) level ≥ 6.5% in national glycohemoglobin standardization program [15]; and dyslipidemia, a serum triglyceride (TG) level ≥ 150 mg/dL, a serum high-density lipoprotein cholesterol (HDL-C) level < 40 mg/dL, or a serum low-density lipoprotein cholesterol (LDL-C) level ≥ 140 mg/dL [16]. To specify the source of aldosterone hypersecretion by AVS, the following diagnostic criteria were used: 1) the laterality ratio (LR) and the contralaterality ratio (CR) calculated before and after the ACTH challenge test in reference to the Japanese guidelines of PA [11]; 2) the absolute PAC value of ≥ 14,000 pg/mL in reference to the articles of Ohmura [17] and Makita [18]; and 3) the aldosterone ratio of the right and left adrenal veins. According to the Japanese guidelines of PA [11], an LR of > 4 and a CR of < 1 after the ACTH challenge test were used as the cutoff values. Tumor laterality was determined based on a CR of < 1 and the absolute PAC value of ≥ 14,000 pg/mL when the ACTH challenge test indicated an LR of 2 to 4 or a discrepancy occurred in tumor laterality before and after the ACTH challenge test. Since serum cortisol levels considerably differed in the adrenal veins of PASCS patients, the adrenal gland secreting cortisol predominantly was determined based on the aldosterone ratio and on the right-to-left ratio of aldosterone and cortisol in the adrenal veins in reference to the article of Hiraishi et al. [8]. Moreover, tumor laterality was determined based on the results from ^131^I-adosterol adrenal scintigraphy and on the absolute value of PAC in reference to the articles of Funder et al. [12] and Minami et al. [13]. We did not measure plasma metanephrine concentrations, although the measurement thereof is useful for determining the need for AVS [19] in patients with the suspected concurrence of aldosterone and cortisol hypersecretion.

### Statistical analyses

Continuous and categorical variables were analyzed according to the one-way analysis of variance and Fisher’s exact test, respectively. Two of the 3 study groups were analyzed according to Student’s t-test. Bonferroni’s correction was applied to the *p* values from Student’s t-test or Fisher’s exact test in multiple comparisons between 2 among the 3 study groups. Blood steroid profiles were compared between 2 groups according to Student’s t-test or the Mann-Whitney U-test.

In addition, the multiple linear regression analysis adjusted for age, sex, and BMI was performed to examine differences in MTD and serum potassium concentration among the PA, SCS, and PASCS groups. MTD was not measured in 1 of 42 patients in the PA group who had a unilateral adrenal tumor. Therefore, the data from the patient were excluded as the missing data.

A value of *p* <  0.05 was considered statistically significant. The JMP software version 9.0 (SAS Institute, Cary, NC, USA) was used to make all statistical analyses except multiple linear regression analysis that was performed using the STATA software version 14 (Stata Corp, College Station, TX, USA).

## Results

### Study population

The clinical, laboratory, and imaging characteristics of 71 patients are shown in Table 1. Mean age was 58.2 ± 11.2 years, females (*n* = 47, 66.2%) were predominant, and mean BMI was 25.2 ± 4.5 kg/m^2^. No significant difference was found in age, sex, and BMI among the PA, SCS, and PASCS groups (Table 1). SBP and DBP of patients with untreated hypertension were 165.6 ± 26.1 mmHg and 96.0 ± 13.6 mmHg, respectively, in the PA group in contrast to 145.6 ± 26.9 mmHg and 80.0 ± 12.7 mmHg, respectively, in the SCS groups. DBP was significantly greater (*p* <  0.01) in the PA group than in the SCS group.

Comorbidities are shown in Table 1. Hypertension occurred in 45 (100%), 9 (75.0%), and 13 (92.9%) patients in the PA, SCS, and PASCS groups, respectively. The proportion of patients with hypertension was significantly greater (*p* <  0.05) in the PA group than in the SCS group; however, no significant difference was found between the PASCS group and the PA group. Notably, the incidence of hypertension was 100% in patients with PA. DM occurred in 6 (14.0%), 6 (50.0%), and 7 (50.0%) patients in the PA, SCS, and PASCS groups, respectively. The proportion of DM patients was significantly greater (*p* <  0.05) in the PASCS group than in the PA group. Dyslipidemia occurred in 25 (56.8%), 10 (83.3%), and 9 (64.3%) patients in the PA, SCS, and PASCS groups, respectively; however, no significant difference was found among these study groups.

Results from laboratory tests are shown in Table 1. FPG was greater not statistically but numerically in the PASCS group than in the PA group (131.6 ± 52.1 mg/dL vs. 103.8 ± 28.5 mg/dL; *p* = 0.09). On the other hand, FPG was statistically greater in the SCS group than in the PA group (150.0 ± 60.7 mg/dL vs. 103.8 ± 28.5 mg/dL; *p* <  0.01). HbA1c was greater not statistically but numerically in the PASCS group than in the PA group (6.5 ± 2.1% vs. 5.7 ± 0.9%; *p* = 0.21). On the other hand, HbA1c was significantly greater in the SCS group than in the PA group (7.3 ± 2.2% vs. 5.7 ± 0.9%; *p* <  0.01). Serum potassium concentration was significantly lower in the PA group than in the SCS group (3.3 ± 0.7 mEq/L vs. 4.0 ± 0.5 mEq/L; *p* <  0.01) and in the PASCS group than in the SCS group (3.2 ± 0.8 mEq/L vs. 4.0 ± 0.5 mEq/L; *p* <  0.01). No significant difference was found in serum potassium concentration between the PA group and the PASCS group. Serum alkaline phosphatase (ALP) level was significantly greater in the PASCS group than in the PA group (279.1 ± 105.4 U/L vs. 212.3 ± 46.3 U/L; *p* <  0.01). No significant difference was found in serum ALP level between the SCS group and the PASCS group.

**Fig. 1:**
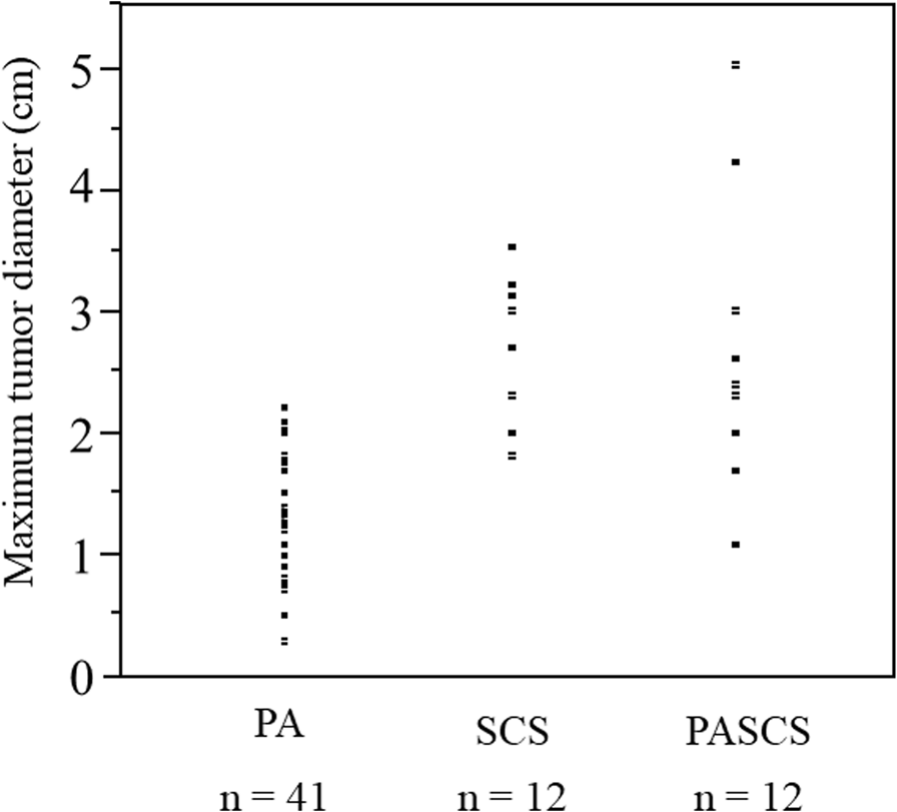
Maximum tumor diameters in patients with PA, SCS, or PASCS who had a unilateral adrenal tumor. PA, primary aldosteronism; SCS, subclinical Cushing’s syndrome, PASCS, primary aldosteronism plus subclinical Cushing’s syndrome

Subsequently, differences in CT Hounsfield units and MTD of adrenal tumors among the 3 study groups were examined with respect to 65 patients who had a unilateral adrenal tumor (Table 2). MTD on the CT scans was significantly greater in the PASCS group than in the PA group (2.7 ± 0.1 cm vs. 1.3 ± 0.1 cm; *p* <  0.001) and was also greater in the SCS group than in the PA group (2.7 ± 0.2 cm vs. 1.3 ± 0.1 cm; *p* <  0.001). No significant difference was found in MTD between the SCS group and the PASCS group. MTD was significantly smaller in the PA group than in the other 2 groups, was second smallest in the SCS group, and was largest in the PASCS group (Table 2). MTD ranged as follows: 0.3–2.2 cm, 1.8–3.5 cm, and 1.1–5.0 cm in the PA, SCS, and PASCS groups, respectively (Fig. 1).

**Table 2:** Maximum tumor diameters and computed tomography Hounsfield units of adrenal tumors in patients who had a unilateral adrenal tumor

	Study groups			*P* values of between-group comparisons			
	PA (*n* = 41^a^)	SCS (n = 12)	PASCS^b^ (n = 12^c^)	All^d^	PA vs. PASCS^e^	SCS vs. PASCS^e^	PA vs. SCS^e^
Image characteristics							
MTD, cm	1.3 ± 0.1	2.7 ± 0.2	2.7 ± 0.1	< 0.0001	< 0.001	NS	< 0.001
Hounsfield number, HU	2.5 ± 19.3	9.2 ± 19.8	11.3 ± 6.6	NS			

Values are expressed as mean ± SD or count (%)

*CT* computed tomography, *PA* primary aldosteronism, *SC* subclinical Cushing’s syndrome, *PASCS* primary aldosteronism plus subclinical Cushing’s syndrome, *MTD* maximum tumor diameter, *HU* Hounsfield unit, *SD* standard deviation, *NS* not significant, *SD* standard deviation

^a^The numbers of patients who remained after the exclusion of the following patients who missed the maximum tumor diameter: 1 patient who had a unilateral adrenal tumor and 3 patients who had bilateral adrenal tumors

^b^Patients with primary aldosteronism plus subclinical Cushing’s syndrome

^c^The numbers of patients who remained after the exclusion of 2 patients who had bilateral tumors and who missed the maximum tumor diameter

^d^Analyzed according to the one-way analysis of variance

^e^Bonferroni’s correction was applied to the *p* values from Student’s t-test or Fisher’s exact test in multiple comparisons between two groups

A value of *p* <  0.05 was considered statistically significant

The blood steroid profiles of patients with PA or PASCS are shown in Table 3. PAC was significantly greater in the PASCS group than in the PA group (255.0 [713.3–153.5] vs. 208.0 [273.0–159.8]; *p* <  0.005). No significant difference was found in PRA in the morning, while the PAC/PRA ratio was significantly greater in the PASCS group than in the PA group (1450.0 [5010.0–529.4] vs. 1258.3 [1956.3–643.1]; *p* <  0.005). The PAC/PRA ratio in the captopril challenge test was significantly greater in the PASCS group than in the PA group (3028.5 ± 3648.9 vs. 730.7 ± 745.7; *p* <  0.001) as with PAC in the captopril challenge test (348.6 ± 340.1 vs. 149.0 ± 94.2; *p* <  0.005). Serum cortisol level was significantly greater in the PASCS group than in the PA group (16.4 ± 6.6 μg/dL vs. 12.4 ± 4.3 μg/dL; *p* <  0.05). The mean serum cortisol level was 17.8 ± 5.9 μg/dL in the SCS group and was not significantly greater in the SCS group than in the PASCS group (17.8 ± 5.9 μg/dL vs. 16.4 ± 6.6 μg/dL; *p* = 0.49). No significant difference was found in plasma ACTH and serum DHEAS levels in the early morning; however, these variables were not significantly lower in the PASCS than in the PA group (*p* = 0.29 for ACTH and *p* = 0.40 for DHEAS). On the other hand, the peak plasma ACTH levels in the CRH challenge test were significantly lower in the PASCS group than in the PA group (18.9 ± 8.9 vs. 57.1 ± 10.8; *p* <  0.005) (Table 3) and were not significantly greater in the SCS group than in the PASCS group (15.3 ± 5.6 μg/dL vs. 18.9 ± 8.9 μg/dL; *p* = 0.64).

**Table 3:** Blood steroid profiles of patients with PA or PASCS

	PA (n = 45)	PASCS (*n* = 14)	PA vs. PASCS
	Mean ± SD Median [IQR]	Mean ± SD Median [IQR]	*P* value
PAC, pg/mL	208.0 [273.0–159.8]	255.0 [713.3–153.5]	< 0.005
PRA, ng/mL/h	0.2 [0.3–0.1]	0.15 [0.3–0.1]	NS
PAC/PRA ratio	1258.3 [1956.3–643.1]	1450.0 [5010.0–529.4]	< 0.005
PRA in the furosemide upright posture challenge test, ng/mL/h	0.4 [0.7–0.2]	0.2 [0.8–0.15]	NS
PAC in the saline infusion test, pg/mL	160.0 ± 166.5	313.5 ± 364.8	NS
PAC/PRA ratio in the captopril challenge test	730.7 ± 745.7	3028.5 ± 3648.9	< 0.001
PAC in the captopril challenge test, pg/mL	149.0 ± 94.2	348.6 ± 340.1	< 0.005
Cortisol, μg/dL	12.4 ± 4.3	16.4 ± 6.6	< 0.05
ACTH, pg/mL	23.1 ± 13.6	18.2 ± 18.8	NS
DHEAS, μg/dL	111.5 ± 75.1	78.5 ± 129.8	NS
ACTH in the CRH challenge test, pg/mL	57.1 ± 10.8	18.9 ± 8.9	< 0.005

Values are expressed as mean ± SD in the upper row or median [IQR] in the lower row

*PA* primary aldosteronism, *PASCS* primary aldosteronism plus subclinical Cushing’s syndrome, *SD* standard deviation, *IQR* interquartile range, *PAC* plasma aldosterone concentration, *PRA* plasma renin activity, *NS* not significant, *ACTH* adrenocorticotropic hormone, *DHEAS* dehydroepiandrosterone sulfate, *CRH* corticotropin-releasing hormone

### Multiple linear regression analysis on MTD and serum potassium concentration with respect to patients in the PA, SCS, and PASCS groups who had a unilateral adrenal tumor

MTD was significantly greater in the PASCS and SCS groups than in the PA group with respect to patients who had a unilateral adrenal tumor (Table 2). Therefore, we conducted a multiple linear regression analysis adjusted for age, sex, and BMI to examine differences in MTD among the PA, SCS, and PASCS groups. Consequently, MTD was significantly smaller in the PA group than in the PASCS group (difference, – 1.19 cm; 95% CI, – 1.66 to – 0.72 cm). However, no significant difference was found in MTD between the SCS group and the PASCS group (Table 4). Serum potassium concentration was significantly greater in the SCS group than in the PASCS group (difference, 0.97 mEq/L; 95% CI, 0.38 to 1.54 mEq/L). However, no significant difference was found in serum potassium concentration between the PASCS group and the PA group (Table 4).

**Table 4:** Multiple regression analysis on maximum tumor diameter and serum potassium concentration with respect to patients in the PA, SCS, and PASCS groups who had a unilateral adrenal tumor (*n* = 65)

	Crude model			Adjusted model^a^		
	Difference	(95% CI)	*P* value	Difference	(95% CI)	*P* value
MTD, cm						
PASCS (*n* = 12^b^)	Reference			Reference		
PA (*n* = 41^c^)	−1.24	(– 1.70 to −0.78)	< 0.001	– 1.19	(– 1.66 to − 0.72)	< 0.001
SCS (n = 12)	−0.04	(– 0.58 to 0.57)	NS	– 0.01	(– 0.55 to 0.54)	NS
Serum potassium concentration, mEq/L						
PASCS (n = 12^b^)	Reference			Reference		
PA (n = 41^c^)	0.23	(−0.23 to 0.69)	NS	0.24	(−0.25 to 0.72)	NS
SCS (n = 12)	0.96	(0.38 to 1.53)	< 0.005	0.97	(0.38 to 1.54)	< 0.005

^a^Consists of 65patients who had a unilateral adrenal tumor (41, 12, and 12 patients with PA, SCS, and PASCS, respectively). All the models were adjusted for age, sex, and BMI at the time of diagnosis

^b^The numbers of patients who remained after the exclusion of 2 patients who had bilateral tumors and who missed the maximum tumor diameter

^c^The numbers of patients who remained after the exclusion of the following patients who missed the maximum tumor diameter: 1 patient who had a unilateral adrenal tumor and 3 patients who had bilateral adrenal tumors

*PA* primary aldosteronism, *SCS* subclinical Cushing’s syndrome, *NS* not significant, *PASCS* primary aldosteronism plus subclinical Cushing’s syndrome, *CI* confidence interval, *MTD* maximum tumor diameter

The cutoff value of 2.4 cm for tumor size seemed to produce the largest proportion of classified patients (91.0%). Patients with PA who had a tumor size of > 2.4 cm almost certainly had the elements of PASCS (specificity 100%). Namely, the sensitivity and specificity were calculated to be 58.0 and 100%, respectively, when the cutoff point for tumor diameter was set to 2.4 cm. The odds ratio for tumor diameter when comparing PA with PASCS was 0.06 (95% CI, 0.006–0.261).

## Discussion

We found several clinical and laboratory differences between patients with PASCS and patients with either PA or SCS. Regarding the impact of PA and SCS on glucose metabolism, the risk of developing DM in SCS is enhanced by the overproduction of cortisol that leads to increased gluconeogenesis [20]. Moreover, the risk is also enhanced by PA through 1) a hypokalemia-induced decrease in initial pancreatic insulin release and 2) a reduction in insulin sensitivity [21–23]. Hypokalemia is caused by the mineralocorticoid receptor-mediated overexcretion of potassium from the kidneys in both hypercortisolism and hyperaldosteronism [12, 24, 25]. Serum potassium concentration decreased significantly in the PA group than in the SCS group (*p* <  0.01). Similarly, the concurrence of PA and SCS significantly decreased serum potassium concentration against the SCS group (*p* <  0.01), but not the PA group. Of special note was the fact that the PASCS group involving both hyperaldosteronism and hypercortisolism did not show any greater decrease in serum potassium concentration as compared with the PA group. The mineralocorticoid receptors (MRs) bind both mineralocorticoids and glucocorticoids with high affinity (deoxycorticosterone = corticosterone ≥ aldosterone = cortisol) [26]. On the other hand, a cortisol-degrading enzyme—11β-hydroxysteroid dehydrogenase type 2 (11β-HSD2)—is expressed in renal epithelial cells and regulates the binding of aldosterone to the MRs by impeding cortisol binding to the MRs through the inactivation of cortisol to cortisone [26, 27]. Namely, this physiological event explains the MR-mediated renal excretion of potassium that is enhanced by both cortisol and aldosterone. We hypothesize that the renal potassium excretion-enhancing activity is greater for aldosterone than for cortisol due to the 11β-HSD2-induced, extensive inactivation of cortisol and that the hyperaldosteronism-enhanced renal excretion of potassium in patients with PASCS becomes more apparent, with the less effect of hypercortisolism on renal potassium excretion. Zallocchi et al. [28] described that renal 11β-HSD2 activity is regulated by glucocorticoids, is downregulated following adrenalectomy, and is restored by corticosterone replacement. These findings lead us to hypothesize that 11β-HSD2 may suppress the binding of corticosteroids to the MRs almost completely in subclinical hypercortisolism or that the expression/activity of renal 11β-HSD2 may be increased in PA. However, these hypotheses require further research for its demonstration.

The proportion of DM patients increased significantly in the PASCS group than in the PA group (*p* <  0.05), which is in line with a previous study that described abnormal glucose metabolism in PA patients with cortisol hypersecretion [29]. Hyperaldosteronism found in patients with PA also induces abnormal glucose metabolism [21–23], although being less intense as compared with hypercortisolism found in patients with SCS. The proportions of DM patients in the PA and SCS groups increased, which resulted to nullify a statistically significant difference in the proportion of DM patients between the 2 study groups. The fact that the risk for DM is increased in PA patients with mild glucocorticoid excess has been reported [30–32]; the finding was also described in Japanese patients with PA and patients with PASCS [33].

Interestingly, patients with PASCS involving hypercortisolism- and hyperaldosteronism-induced hypokalemia showed neither additive or synergic impact on abnormal glucose metabolism contrary to our prediction. The proportion of DM patients was comparable between the PASCS group and the SCS group. However, the reason for these findings is unknown, awaiting the further accumulation of clinical evidence.

MTD was significantly smaller (*p* <  0.001) in the PA group than in the PASCS or SCS group, and multiple regression analysis on MTD revealed that MTD was significantly larger by 1.2 cm in the PASCS group than in the PA group (*p* <  0.001). Previous studies [8, 34] examined the clinical characteristics of patients with PA or PASCS and described significant differences in MTD between the 2 study groups. Their results were concordant with and support our results that indicated no significant difference in MTD between the PASCS group and the SCS group.

Most of previous clinical studies in patients with SCS have described adrenal tumors of ≥ 2 cm in diameter [35, 36]. In addition, an adrenal adenoma causing the overproduction of both cortisol and aldosterone is considered to have a ≥ 2.5 cm diameter [34]. In the present study, however, the adrenal tumor was smaller in both patients with SCS and patients with PASCS. Concretely, the smallest MTD was 1.1 cm in patients with PASCS (Fig. 1). None of patients, who had PA and an adrenal tumor < 1 cm in diameter, developed SCS. Therefore, the dexamethasone suppression test may not be required for them.

Regarding bone metabolism impairment in SCS, the risk of developing osteoporosis is enhanced by the overproduction of cortisol in SCS [37, 38]. On the other hand, hyperaldosteronism is also known to increase the risk for osteoporosis [39]. SCS and PA are the risk factors for a reduction in BMD and an increase in vertebral fracture [37–39]. In the present study, serum ALP level was significantly greater in the PASCS group than in the PA group (*p* <  0.01). No significant difference was found in serum ALP level between the SCS group and the PASCS group. If this ALP represents bone alkaline phosphatase (BAP), the deleterious effects of hyperaldosteronism on bone metabolism might be masked by the severe abnormalities of bone metabolism caused by hypercortisolism in patients with PASCS. However, the relevant effects are difficult to assess by means of bone metabolism markers [eg, BAP] in patients with hypercortisolism as found in SCS [37]. Unfortunately, we neither used bone metabolism markers, nor measured BMD. Therefore, we will intend to investigate these variables in the future.

### Limitations

The present study has several limitations. First, the study was retrospective in design and had a relatively small number of patients. Therefore, selection bias and sampling bias cannot be discarded. Second, not all patients underwent AVS or had a histopathological diagnosis. Patients, to whom challenge tests for either PA or SCS were conducted, were not included in the present study. Hence, the number of patients resulted to be relatively small. Third, the lack of data in the present study impeded the analysis of BMD and bone metabolism markers. Fourth, ^131^I-adosterol adrenal scintigraphy is not only useful for the diagnosis of SCS, but also is a very important imaging modality to predict postsurgical hypoadrenalism [40]. However, we could not investigate the latter.

## Conclusions

We could not obtain any reference criteria to surely distinguish patients with concurrent endocrinopathies from those with a single endocrinopathy. However, clinicians should suspect the presence of concurrent SCS in patients with PA when detecting an adrenal tumor (≥ 1 cm in diameter) on the CT scans.

## Data Availability

The datasets analyzed during the current study are available from the corresponding author on a reasonable request.

## Abbreviations

ACTH: Adrenocorticotropic hormone
ALP: Alkaline phosphatase
BMI: Body mass index;
CRH: corticotropin-releasing hormone
CT: computed tomography
DBP: Diastolic blood pressure
DHEAS: Dehydroepiandrosterone sulfate
FPG: Fasting plasma glucose
HbA1c: Hemoglobin A1c
HDL-C: High-density lipoprotein cholesterol
HU: Hounsfield unit
LDL-C: Low-density lipoprotein cholesterol
MTD: Maximum tumor diameter
NGSP: National glycohemoglobin standardization program
PA: Primary aldosteronism
PAC: Plasma aldosterone concentration
PASCS: Primary aldosteronism plus subclinical Cushing’s syndrome
PRA: Plasma renin activity
SBP: Systolic blood pressure
SCS: Subclinical Cushing’s syndrome
TG: Triglyceride
UA: Uric acid
